# Effects of Esketamine on Post-Partum Depression in Patients With Different Personality Types Undergoing Caesarean Section: Randomised Controlled Trial

**DOI:** 10.62641/aep.v53i4.1965

**Published:** 2025-08-05

**Authors:** Mei Luo, Ni Tang, Yang Guo, Di Fan, Xiaobin Wang

**Affiliations:** ^1^Department of Anesthesiology, The Affiliated Hospital of Southwest Medical University, 646000 Luzhou, Sichuan, China

**Keywords:** esketamine, personality, caesarean section, post-partum depression

## Abstract

**Background:**

As an intravenous general anaesthetic, esketamine has rapid and evident antidepressant effects and therefore helps prevent post-partum depression (PPD). This study aimed to observe the effect of intraoperative esketamine application on patients with PPD undergoing caesarean section and to explore whether this effect varies among patients with different personality types.

**Methods:**

A total of 280 patients who underwent elective caesarean section under spinal anaesthesia were randomly divided into esketamine and control groups. On the day before the surgery, each patient was assessed using the Edinburgh Post-partum Depression Scale (EPDS), Self-Rating Anxiety Scale(SAS), Self-Rating Depression Scale (SDS) and Eysenck Personality Questionnaire. Additionally, the pressure–pain threshold was measured. The esketamine group received a single intravenous injection of esketamine at a dose of 0.25 mg/kg (diluted to 5 mL and administered intravenously within 10 min after foetus removal). The control group received 5 mL of 0.9% normal saline. The primary outcome was PPD incidence, assessed using the EPDS on the 3rd post-operative day. The secondary outcomes included post-operative pain score and esketamine safety assessment.

**Results:**

Statistically significant differences in PPD incidence were observed among patients with different personality types (introverted unstable, 66.70%; extroverted unstable, 45.50%; extroverted stable, 19.40%; and introverted stable, 15.00%, *p *< 0.05). The patients with an extroverted-stable personality in the esketamine group had a lower PPD incidence than those in the control group (11.90% vs. 25.70%, *p *< 0.05). No statistical difference in total PPD incidence was observed between the two groups (35.7% vs. 29.3%, *p *> 0.05). Pain scores in the esketamine group were lower than those in the control group while at rest (4, 24 and 48 h) and during movement (4 and 8 h) after surgery (*p *< 0.05). The mean arterial pressure and heart rate in the esketamine group were higher than those in the control group during surgery (*p *< 0.05).

**Conclusion:**

A single intravenous administration of esketamine had no apparent effect on the overall PPD incidence among patients undergoing caesarean section. It may have a beneficial effect in reducing PPD incidence in patients with an extroverted-stable personality.

**Trial Registration:**

Chinese Clinical Trial Registry, ChiCTR2100050976, 09/09/2021, http://www.chictr.org.cn.

## Introduction

Post-partum depression (PPD) refers to mood and behavioural disturbances associated with the puerperium and typically occurring within 4 weeks post-partum. Symptoms include post-partum sleep disorders, anxiety and irritability [1, 2]. The *Diagnostic and Statistical Manual of Mental Disorders, Fifth Edition* [3] classifies PPD as a major depressive disorder (MDD) with peripartum onset, defined as depressive episodes emerging during pregnancy or within 4 weeks after delivery. Cases not fully meeting MDD criteria but exhibiting a recent major depressive episode are also included. The *International Classification of Diseases* offers similar diagnostic criteria but extends the timeframe to 6 weeks post-partum. However, studies reported PPD symptoms as early as 10 days post-partum or as late as 3, 6, 12 and 36 months after delivery [4, 5, 6]. PPD is not a homogeneous condition but encompasses multiple disease pathways. Clinical diagnosis and research should distinguish its timing of onset and association with childbirth [7]. Evidence suggests that the aetiology of PPD is complex and closely related to maternal physical, psychological, social and genetic factors [8, 9]. The importance psychosocial factors in increasing PPD risk has been widely recognised. The International Association of Perinatal Mental Health recommends evaluating the role of personality traits in PPD occurrence and development. Neuroticism is the most widely studied personality trait; individuals with this personality trait may experience great stress during pregnancy and childbirth. It is one of the most important predictors of PPD [10, 11] and increases the risk of PPD and suicidal ideation [12, 13]. Owing to differences in research methods, diagnostic criteria and demographic characteristics in different countries and regions, the reported incidence of PPD varies greatly. A meta-analysis of 32,307 Asian women found that the PPD prevalence in this population was 3.5%–63.3%, with the lowest in Malaysia (3.9%) and the highest in Pakistan (63.3%) [14]. Among the related psychological factors, personality traits play an important role in PPD onset [10, 11, 15, 16].

PPD is harmful to mothers and has many adverse effects on infants and family members [17, 18, 19]. Psychological interventions and medications are the primary preventive and therapeutic measures against PPD. Cognitive behavioural therapy and interpersonal psychotherapy are the most common psychological interventions [20]; and anxiolytics, tricyclic antidepressants and norepinephrine reuptake inhibitors are the most common therapeutic drugs [21].

As an intravenous general anaesthetic drug, esketamine has rapid and evident antidepressant effects. The US Food and Drug Administration has approved a nasal spray of esketamine for the clinical treatment of major depressive disorders [22]. A single intravenous infusion of a subanaesthetic dose of esketamine produces rapid (within 2 h) and significant antidepressant effects [23]. As an anaesthetic drug with rapid antidepressant and pre-emptive analgesic effects, esketamine could be used perioperatively to prevent PPD. However, the effect of a single intravenous dose of esketamine on the depression of patients after caesarean section under spinal anaesthesia remains unknown. Therefore, this study investigated the effect of esketamine on PPD to provide a basis for its clinical use in the prevention and treatment of this disease.

## Methods

### Study Design

This single-centre, prospective, double-blind, randomised controlled trial was approved by the Ethics Committee of the Affiliated Hospital of the Affiliated Hospital of Southwest Medical University (KY2021299) and registered at http://www.chictr.org.cn (ChiCTR2100050976; 09/09/2021). The study protocol strictly conformed to the CONSORT guidelines.

### Participants

From January 15 to July 30, 2022, patients scheduled for elective caesarean section under spinal anaesthesia were enrolled in this study after signing a written informed consent form. The inclusion criteria were as follows: (1) a normal singleton pregnancy with a gestation period of at least 37 weeks; (2) American Society of Anesthesiologists (ASA) class II; (3) body mass index (BMI) of 18.5–35 kg/m^2^; and (4) age of 18–40 years. The exclusion criteria were as follows: (1) refusal to sign the consent form; (2) contraindications to spinal anaesthesia; (3) history of opioid abuse or tolerance, related drug allergies or recent use of antipsychotics; (4) inability to complete any scale in this study on their own; (5) pregnancy complications, such as aggravated placenta previa, gestational hypertension, eclampsia, pre-eclampsia, intrauterine distress, intrauterine infection or stillbirth; (6) severe cardiopulmonary disease or severe hepatic or renal function insufficiency; and (7) Lie scale score of >60 in the Eysenck Personality Questionnaire (EPQ) after analysis (indicating doubtful authenticity). The following mid-test exclusion criteria were used: (1) intraoperative changes to general anaesthesia for various reasons; (2) serious complications such as intraoperative or post-operative haemorrhage or amniotic fluid embolism; (3) neonatal deaths; and (4) no post-operative follow-up.

### Randomisation and Blinding

On the basis of the random sequences generated using Microsoft Excel (Microsoft Office Excel 2007 (Version 12.0); Microsoft Corp, USA, https://office.microsoft.com/excel), all the participants were randomised 1:1 to an esketamine or a control group. The grouping information was concealed in sequentially coded, sealed, opaque envelopes (two identical envelopes were prepared, and the envelope code was the group number). One sealed opaque envelope containing the study number was opened by an anaesthetist who was not involved in the study before surgery. The other identical envelope was kept by the principal investigator and opened only upon completion of data collection at the end of the study. The same anaesthetist prepared the study medication and provided it to an anaesthetist responsible for intraoperative interventions, who then recorded ‘intervention given’ in the anaesthesia record. Post-operative pain score and PPD assessment were performed independently by two anaesthetists. The anaesthetists involved in the study (except those responsible for intraoperative administration), patients and other healthcare providers involved in post-operative care were blinded to the grouping. Psychologists from the Affiliated Hospital of Southwest Medical University supervised this study and trained the researchers who conducted the scale evaluation and recorded the results.

### Study Protocol

All the patients included in the study were assessed using the Edinburgh Post-partum Depression Scale (EPDS), Eysenck Personality Questionnaire (EPQ), Self-Rating Depression Scale (SDS) and Self-Rating Anxiety Scale (SAS) the day before surgery. After being informed of the requirements of each scale, the patients answered the questions independently on a written form. The EPDS [24] consists of 10 questions, each rated on a four-point scale based on the frequency of symptoms (0–3). EPDS ≥10 was used as the criterion for PPD [25], with a Cronbach’s coefficient of 0.76 and a content validity of 0.90. The EPQ has been widely used to evaluate personality characteristics. This study adopted the Chinese version of the EPQ revised by Gong [26] with a Cronbach’s coefficient of 0.78 for reliability and validity. This questionnaire has 88 questions and includes four scales: introversion–extroversion (E), neuroticism (N), psychoticism (P) and lying (L). The first three represent the three personality dimensions, and L is a validity scale. The options for each question were ‘yes’ and ‘no’. For positively scored questions, ‘no’ is scored as 0 and ‘yes’ as 1; and the opposite is true for negatively scored questions. Personality types were identified using the E dimension as the X-axis and the N dimension as the Y-axis, intersecting at T50. The participants with low and high E scores were classified as introverts and extroverts, respectively; and those with low and high N scores were classified as stable and unstable, respectively. The following four personality types were identified: introverted unstable, extroverted unstable, extroverted stable and introverted stable. The SAS [27] consists of 20 questions reflecting a range of self-perceptions related to anxiety, each rated on a four-point scale based on the frequency of symptoms (1–4). Its Cronbach’s coefficient is 0.931 for reliability and validity. The SDS [28] consists of 20 questions reflecting a range of self-perceptions related to depression, each rated on a four-point scale based on the frequency of symptoms (1–4). Its Cronbach’s coefficient is 0.863. Pressure–pain threshold was measured by placing a handheld manometer (Wagner instruments FDX 25, USA) at the thenar muscle of the right palm of each patient the day before the procedure; the average of three consecutive measurements was calculated. The probe was maintained perpendicular to the measurement area while slowly and evenly applying pressure and moved slightly during each measurement to avoid the continuous measurement of the same area. Scale assessments and pain threshold measurements were performed independently by two professionally trained anaesthetists. Demographic and basic data, including age, BMI, education, employment, marital status, previous pregnancies, previous deliveries, previous caesarean sections and pregnancy complications, were collected from all the participants.

All the patients underwent surgery under spinal anaesthesia, and their blood pressure, electrocardiogram and pulse oxygen saturation were monitored immediately after entering the operating room. All the patients were administered with 500 mL of lactated Ringer’s solution prior to anaesthesia (Sichuan Kelun Pharmaceutical Company,China No: H20055488). The puncture was performed at L_3-4_ in the left lateral position. After successful puncture and free cerebrospinal fluid flow, 3 mL of 0.67% ropivacaine was injected (Ruiyang Pharmaceutical Company, China, No: H20183152). The aim of the block was to achieve a sensory level above T6. The vital signs of the patients were routinely monitored (recorded every 5 min). Vasoactive drugs were administered depending on the patient’s blood pressure and heart rate (HR) to maintain stable vital signs (prophylactic administration of vasoactive drugs to ensure blood pressure stability when blood pressure showed a downward trend). Patient-controlled intravenous analgesia consisting of 0.75 µg/mL sufentanil citrate (Yichang Renfu Pharmaceutical Company, China, No: H20054171), 0.05 mg/mL butorphanol (Jiangsu Hengrui Pharmaceutical Company, China, No: H20020454) and 0.045 mg/mL granisetron (Sichuan Taiji Pharmaceutical Company, China, No: H20030161) in 200 mL saline was provided to all the patients after the surgery. The infusion rate was 3 mL/h, and the lockout time was 15 min. The caesarean sections were performed by the same team of surgeons.

Within 10 min after foetal removal, the patients in the esketamine group were intravenously injected with 0.25 mg/kg esketamine (Jiangsu Hengrui Pharmaceutical Company, China, No: H20193336) (diluted to 5 mL with 0.9% saline), whereas those in the control group received 5 mL 0.9% saline (Sichuan Kelun Pharmaceutical Company, China, No: H51020633). HR and mean arterial pressure (MAP) were recorded at the time of administration, 5–30 min after administration (recorded every 5 min) and before the patient left the operating room. Ramsay sedation scores (RSS) were recorded 5, 10 and 15 min after administration and assessed using the following criteria: score 1, anxious; score 2, awake, quiet and cooperative; score 3, drowsy but responsive to commands; score 4, light sleep and could be awakened quickly; score 5, asleep and does not respond to loud calls; and score 6, deep sleep and does not respond to stimuli. Any complications following medication administration, such as dizziness, nausea, vomiting, dyspnoea, hallucinations and nightmares were recorded. All complications were assessed and recorded after the patients were awakened. Blood loss, duration of surgery, post-operative vaginal bleeding and newborn sex were also recorded.

The numeric rating scale (NRS) was used to evaluate the analgesic effect at 4, 8, 12, 24 and 48 h post-surgery. Pain at rest and during movement was evaluated at these time points. Raising the legs was considered a movement at 4 and 8 h post-operatively, and autonomous turning was considered a movement at 12, 24 and 48 h post-operatively. The NRS assesses pain using numbers between 0 and 10 to indicate the pain level (0, none; 1–3, mild; 4–6, moderate; and 7–10, severe). Additionally, the following variables were measured and documented: vaginal bleeding 6 h post-operatively, post-operative indomethacin use, neonatal status and hospital stay.

### Outcome Measures

The primary outcome was PPD incidence. A PPD diagnosis is typically made when the EPDS score is ≥10 [3, 25]. The secondary outcomes included the post-operative analgesic effect of esketamine and its safety, which was evaluated in terms of intraoperative vital signs, intraoperative and post-operative complications and RSSs.

### Sample Size Calculation

Observational studies in China using EPDS as an assessment tool for PPD and an EPDS score of ≥10 as the diagnostic criterion reported a 28% PPD incidence on the 3rd day after a caesarean section [29]. With 80% power and a 5% level of significance (two-sided), the calculated sample size included a 10% dropout rate, requiring 288 participants to detect a reduction of 50% in the incidence of PPD. The sample size was calculated using the Power Analysis and Sample Size software [PASS 15 (2017); NCSS, LLC. Kaysville, Utah, USA, ncss.com/software/pass]. The specific calculation formula is as follows:

$$n_{1}=n_{2}={\left[\frac{Z_{\alpha /2}\,\sqrt{2\,\pi_{c}\,\left(1-\pi_{c}\right)}+Z_{\beta }\,\sqrt{\pi_{1}\,\left(1-\pi_{1}\right)+\pi_{2}\,\left(1-\pi_{2}\right)}}{\pi_{1}-\pi_{2}}\right]}^{2},\pi_{c}=\frac{\pi_{1}+\pi_{2}}{2},$$

where π_1_ and π_2_ represent the assumed PPB incidence rates in the two groups.

### Statistical Analysis

All statistical analyses were performed using SPSS version 25 (IBM Corp. Released 2017. IBM SPSS Statistics for Windows, Version 25.0. Armonk, NY: IBM Corp). Categorical data were presented as frequencies and percentages and analysed using χ^2^ or Fisher exact tests. Continuous variables were presented as means and standard deviations (SD) if they were normally distributed or as medians and quartiles if not. Normality was verified through Shapiro–Wilk tests with graphical confirmation via P-P/Q-Q plots. Groups were compared using *t* tests if they were normally distributed or the Mann–Whitney test if not. HR and MAP were examined by analysis of variance for repeated measurements. Subgroup analyses were performed according to personality type. All statistical tests were two-sided, and a *p* value (adjusted by Bonferroni) < 0.05 was considered statistically significant.

## Results

### Participants’ Characteristics

**Fig. 1.**
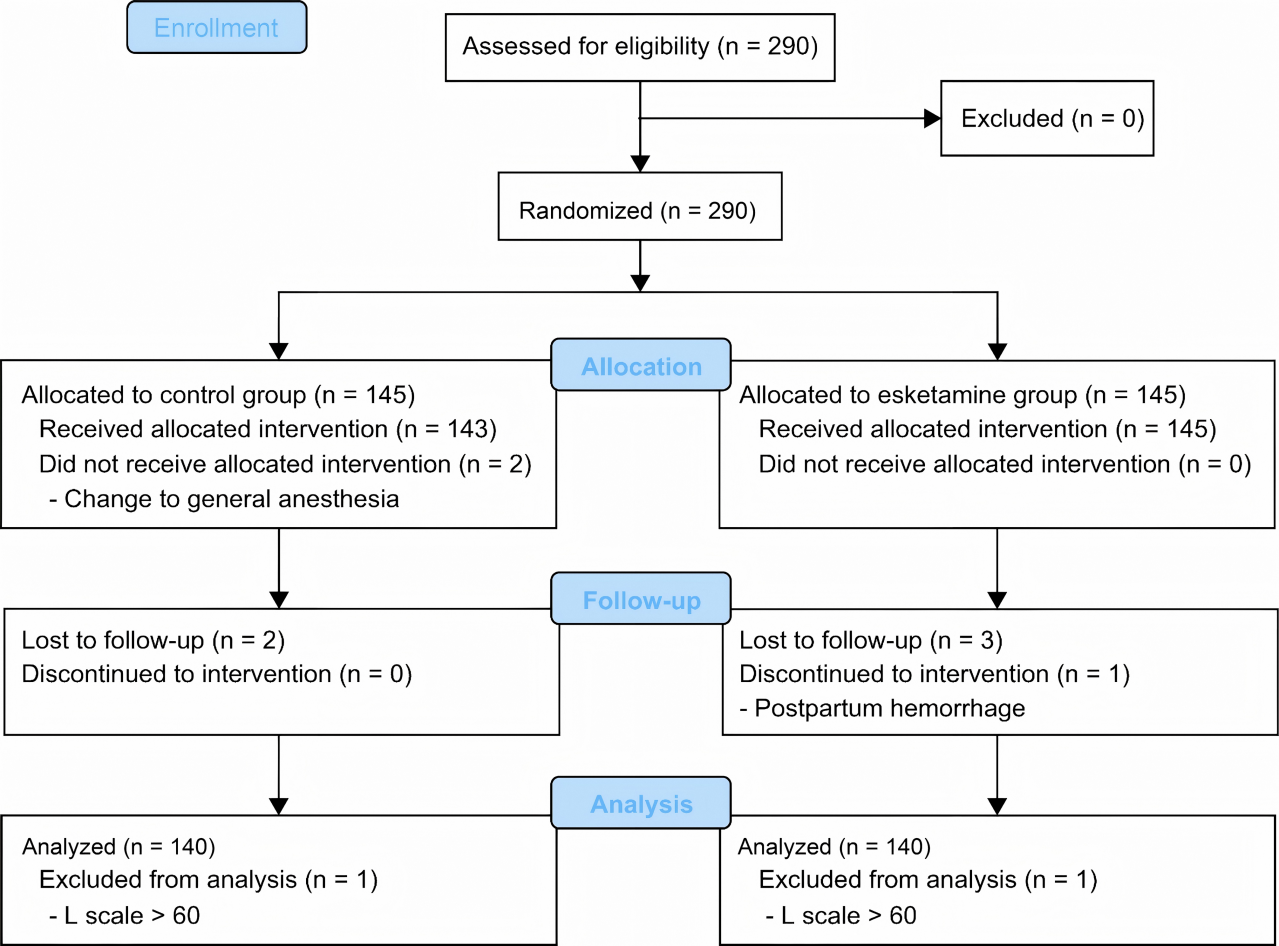
**Study flow chart**.

A total of 290 participants were recruited for this study. Two participants were excluded due to changing the anaesthesia method to general anaesthesia intraoperatively, one was excluded due to post-partum haemorrhage, five were excluded because they were lost to follow-up and two were excluded due to having L-scale scores >60 (Fig. 1). The baseline characteristics of age, BMI and gravidity in the two groups are shown in Table 1. According to the EPQ scores in the pre-operative assessment, personality was classified into four types: introverted stable (40 patients), introverted unstable (45 patients), extroverted stable (129 patients) and extroverted unstable (66 patients) (Table 1).

**Table 1.** **Baseline data of patients in the two groups**.

		Control group (n = 140)	Esketamine group (n = 140)	t/χ²/Z	*p* value
Age (years)		30.1 ± 4.9	30.1 ± 4.2	0.837	0.457
BMI (kg/m^2^)		28.3 ± 3.3	27.9 ± 2.9	0.936	0.350
Gravidity		2 (1, 3)	2 (1, 4)	−1.064	0.287
Parity		0 (0, 1)	1 (0, 1)	−1.541	0.123
Number of previous CS		0 (0, 1)	0 (0, 1)	−1.326	0.185
Employment, ո (%)				0.753	0.386
	Unemployed	55 (39.3)	48 (34.3)		
	Employed	85 (60.7)	92 (65.7)		
Education level, ո (%)				1.902	0.386
	Secondary school	35 (25.0)	36 (25.7)		
	High school	19 (13.6)	27 (19.3)		
	University	86 (61.4)	77 (55.0)		
Marital status, ո (%)					1.000
	Unmarried	4 (2.9)	5 (3.6)		
	Married	136 (97.1)	135 (96.4)		
Pregnancy complications^&^, ո (%)		46 (32.9)	53 (37.9)	1.695	0.428
Duration of surgery (min)		55 (48, 65)	58 (50, 70)	−1.716	0.086
Blood loss (mL)		300 (300, 400)	300 (300, 400)	−0.812	0.417
Length of hospital stay (days)		4 (4, 4)	4 (4, 4)	−0.039	0.969
Post-operative oxytocin, ո (%)		38 (27.1)	47 (33.6)	1.368	0.242
Newborn’s gender, ո (%)				0.229	0.632
	Boy	74 (53)	70 (50)		
	Girl	66 (47)	70 (50)		
Newborn in NICU, ո (%)		21 (15.0)	15 (10.7)	1.148	0.284
Pain threshold (lbf)		6.2 ± 1.7	6.4 ± 1.7	−0.843	0.400
Pre-operative SAS score		31.2 ± 6.3	31.2 ± 6.8	−0.018	0.986
Pre-operative SDS score		45.7 ± 10.1	45.8 ± 9.0	−0.039	0.969
Pre-operative EPDS score		9.3 ± 4.0	8.9 ± 4.0	0.818	0.414
Prenatal depression, ո (%)		64 (45.7)	57 (40.7)	0.713	0.398
Personality types, ո (%)				2.753	0.431
	Introverted stable	21 (15)	19 (14)		
	Introverted unstable	21 (15)	24 (17)		
	Extroverted stable	70 (50)	59 (42)		
	Extroverted unstable	28 (20)	38 (27)		

BMI, Body mass index; CS, Caesarean section; NICU, Neonatal care unit; SAS, Self-rating anxiety scale; SDS, Self-rating depression scale; EPDS, Edinburgh post-partum depression scale. ^&^Pregnancy complications include only gestational diabetes or gestational hypothyroidism.

### PPD

The patients with extroverted-stable personality in the esketamine group had a lower incidence of PPD than those in the control group (11.9% vs. 25.7%, *p* < 0.05). However, no overall statistical difference in total PPD incidence was observed between the two groups (35.7% vs. 29.3%, *p* > 0.05) (Table 2).

**Table 2.** **Incidence of post-partum depression**.

		Control group (n = 140)	Esketamine group (n = 140)	χ *²*	*p* value
PPD (total), ո (%)		50 (35.7)	41 (29.3)	1.319	0.251
With different personalities					
	Introverted stable	4 (19.0)	2 (10.5)		0.664
	Introverted unstable	13 (61.9)	17 (70.8)	0.402	0.526
	Extroverted stable	18 (25.7)	7 (11.9)	3.931	0.047*
	Extroverted unstable	15 (53.6)	15 (39.5)	1.292	0.256

PPD, Post-partum depression; *p value < 0.05. Fisher exact test without χ^2^ value.

### Post-Operative Analgesic Effect

**Fig. 2.**
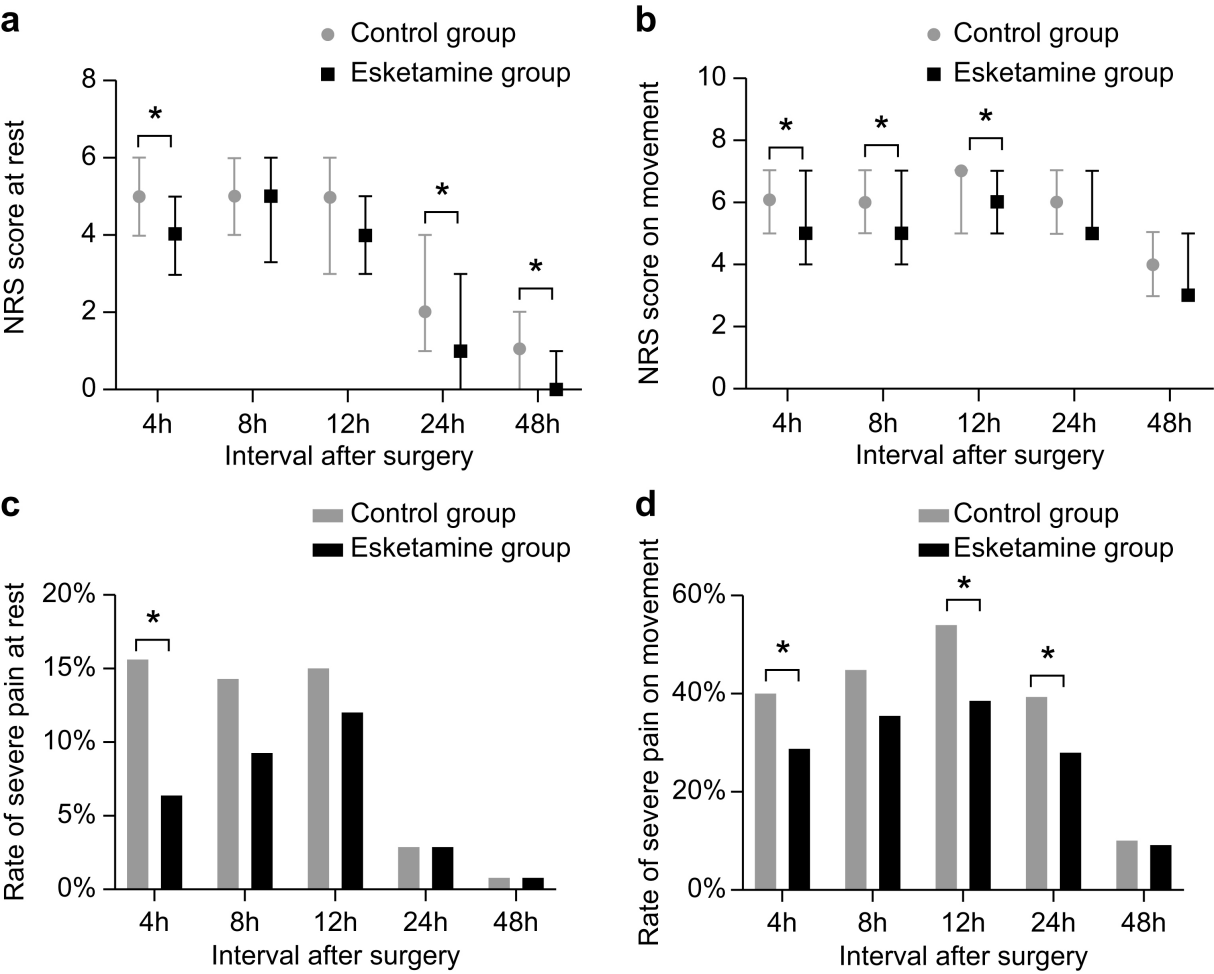
**Post-operative analgesic effect: pain score and rate of severe pain during different time intervals after surgery**. Numeric rating scale scores at rest (a) and on movement (b). Rate of severe pain at rest (c) and on movement (d). *p value < 0.05. NRS, numeric rating scale. Definition of severe pain: NRS score ≥7.

Compared with those in the control group, the NRS scores while at rest (4, 24 and 48 h) and during movement (4, 8 and 12 h) were lower in the esketamine group (*p* < 0.05). No statistically significant differences were observed between the two groups at other time intervals (Fig. 2a,b). NRS ≥7 was used as the criterion for severe pain to investigate the effect of esketamine on severe pain. The incidence of severe pain while at rest (4 h) and during movement (4, 12 and 24 h) was lower in the esketamine group (*p* < 0.05) than in the control group. No statistically significant difference was found between the two groups at other time intervals (Fig. 2b–d).

**Fig. 3.**
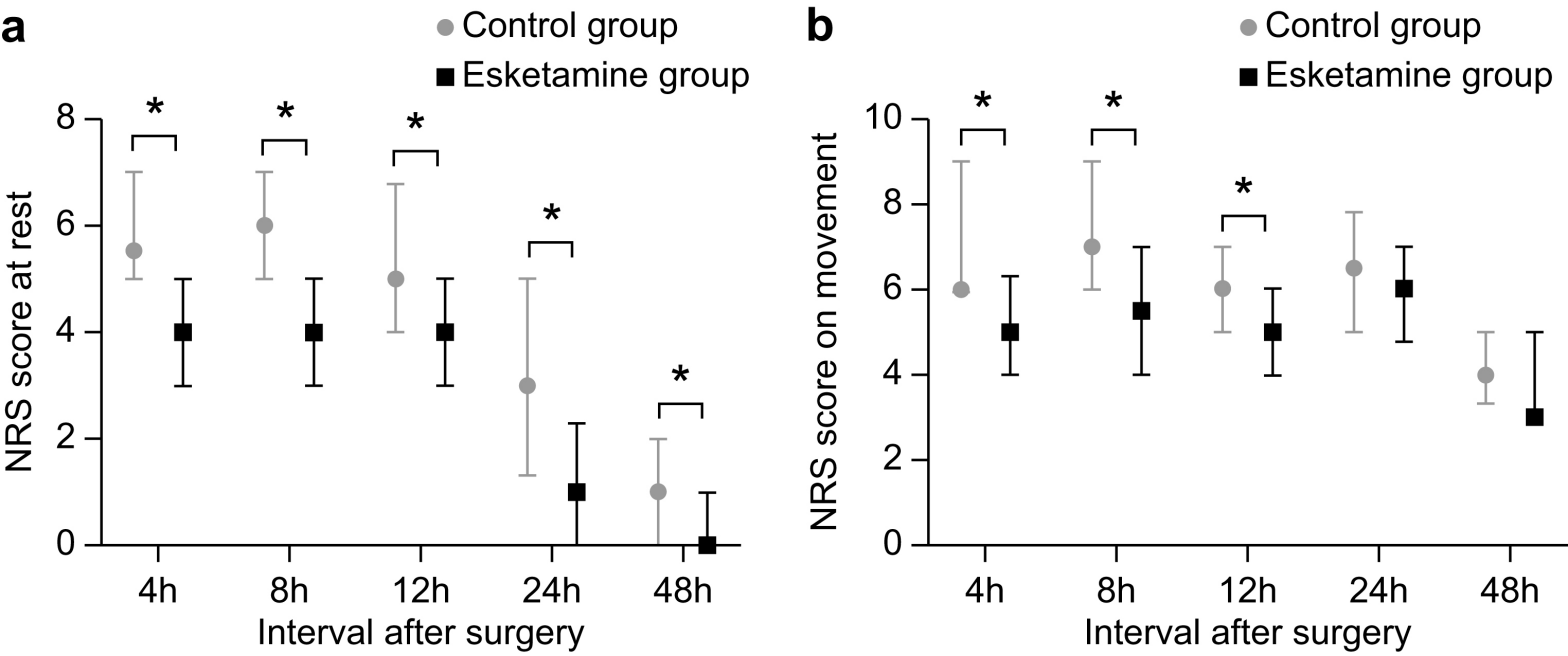
**Numeric rating scale scores at rest (a) and on movement (b) during different time interval after surgery in patients with an extroverted-unstable personality**. *p value < 0.05. NRS, numeric rating scale.

Furthermore, the post-operative analgesic effect of esketamine differed among the personality types. Among the patients with an extroverted-unstable personality, those in the esketamine group had lower NRS scores than those in the control group while at rest (4, 8, 12, 24 and 48 h) and during movement (4, 8 and 12 h, *p* < 0.05) (Fig. 3a,b). However, no statistically significant difference was observed between the two groups regarding the other three personality types (**Supplementary Fig. 1**).

### PPD Among Different Personality Types

The numbers and incidence rates of PPD in patients with different personality types were as follows: 6 and 15.0% for those with an introverted-stable personality, respectively; 30 and 66.7% for those with an introverted-unstable personality, respectively; 25 and 19.4% for those with an extroverted-stable personality, respectively; and 30 and 45.5% for those with an extroverted-unstable personality, respectively. Statistically significant differences in the number and incidence rate of PPD were found among the different personality types (*p* < 0.05). Similar differences were observed for the incidence of prenatal depression and pre-operative SDS and SAS scores (*p* < 0.05). However, no statistically significant differences were noted for pain thresholds (Table 3).

**Table 3.** **Data for different personality types**.

	Introverted-	Introverted-	Extroverted-	Extroverted-	*p*-value
	stable (n = 40)	unstable (n = 45)	stable (n = 129)	unstable (n = 66)	
PPD, ո (%)	6 (15.0)^b,d^	30 (66.7)^a,c^	25 (19.4)^b,d^	30 (45.5)^a,c^	<0.001
Prenatal depression, ո (%)	10 (25.0)^b,d^	36 (80.0)^a,c^	32 (24.8)^b,d^	43 (65.2)^a,c^	<0.001
Pre-operative SAS score	28.88 ± 5.65^b,d^	35.24 ± 6.34^a,c^	28.77 ± 5.50^b,d^	34.42 ± 6.5^a,c^	<0.001
Pre-operative SDS score	43.41 ± 8.86^b,d^	53.06 ± 7.34^a,c^	42.28 ± 8.78^b,d^	49.03 ± 8.95^a,c^	<0.001
Pre-operative Pain threshold (lbf)	5.92 ± 1.56	6.41 ± 1.68	6.37 ± 1.65	6.33 ± 1.76	0.473

PPD, post-partum depression; SAS, self-rating anxiety scale; SDS, self-rating depression scale. Vs introverted-stable personality, ^a^*p* < 0.05; vs introverted-unstable personality, ^b^*p* < 0.05; vs extroverted-stable personality, ^c^*p* < 0.05; vs extroverted-unstable personality, ^d^*p* < 0.05.

### Esketamine’s Safety

**Fig. 4.**
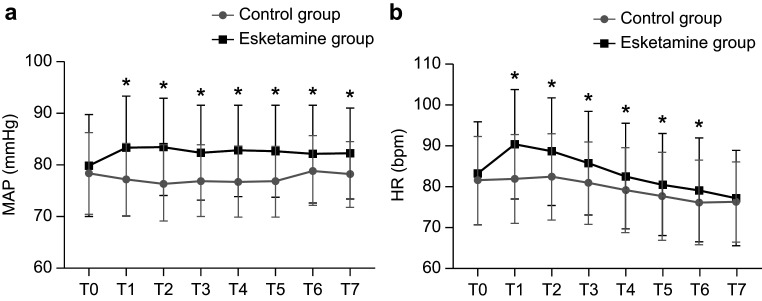
**Mean arterial (a) and heart rate (b) pressure during different time interval after surgery**. *p value < 0.05. MAP, mean arterial; HR, heart rate.

Statistically significant differences were observed in the trends of MAP and HR from the time of administration to the time of leaving the operating room in both groups (*p* < 0.05). MAP was higher in the esketamine group than in the control group at 5 (T1), 10 (T2), 15 (T3), 20 (T4), 25 (T5) and 30 min (T6) and after leaving the operation room (T7). Similarly, HR was higher in the esketamine group than in the control group at all time points except at T7 (Fig. 4).

For the complications after esketamine administration, no statistically significant differences were found in the incidences of dizziness, nausea and vomiting, hallucinations and nightmares between the two groups intra- and post-operatively. The incidence rates of RSS >3 in the esketamine group at 5 and 10 min after administration were 82.9% and 14.3%, respectively, which were significantly higher than those in the control group (*p* < 0.001). However, no statistical difference was observed at the time point of 15 min after administration or when leaving the room (Table 4).

**Table 4.** **Adverse reactions associated with esketamine**.

			Control group (n = 140)	Esketamine group (n = 140)	χ²	*p* value
Adverse reactions, ո (%)						
	Intra-operative					
		Dizziness	0 (0.0)	4 (2.9)		0.122
		Nausea and vomiting	2 (1.4)	5 (3.6)		0.447
		Hallucinations	0 (0.0)	2 (1.4)		0.498
		Nightmares	0 (0.0)	5 (3.6)		0.06
	Post-operative					
		Dizziness	4 (2.9)	1 (0.7)		0.370
		Drowsiness	19 (13.6)	16 (11.4)	0.294	0.588
		Nausea and vomiting	2 (1.4)	6 (4.3)		0.161
RSS score (>3), ո (%)						
After administration 5 min			3 (2.1)	116 (82.9)		<0.001*
After administration 10 min			0 (0)	20 (14.3)		<0.001*
After administration 15 min			0 (0)	3 (2)		0.247
Leaving the operating room			0 (0)	0 (0)		

RSS, Ramsay sedation scores. *p value < 0.05. Fisher exact test without χ^2^ value.

## Discussion

In this study, we found that the patients with an extroverted-stable personality in the esketamine group had a lower PPD incidence than those in the control group. However, no overall statistical difference in total PPD incidence was observed between the two groups.

PPD is caused by a variety of factors, including education, employment, lack of social support, poor life quality, poor family relationships, personality, history of depression, premenstrual syndrome and acute pain after a caesarean section, all of which are risk factors of this disease [9, 15, 30, 31]. Therefore, we evaluated PPD on the 3rd day after surgery because it is the best time to observe the effect of esketamine and it is when the interference of family, society, children and other factors on the mother is minimal [32]. Moreover, early screening can reduce the incidence of depression and suicide. In this study, the BMI and NRS scores for movement (at 8 and 48 h) after the operation, neuroticism, psychoticism and prenatal depression were found to be associated with PPD. Although the pain scores in the esketamine group were significantly lower than those in the control group during movement (at 4 and 8 h) and at rest (at 4, 24 and 48 h), post-operative analgesia improved to some extent. However, owing to the complex mechanisms and numerous risk factors of PPD, its incidence was not reduced by improving a single factor.

This study also found that a single intravenous injection of 0.25 mg/kg esketamine did not reduce the incidence of early post-operative PPD, which may be related to the dosage and timing of administration. The antidepressant mechanism of esketamine is currently unclear; however, increased serum BDNF levels are considered the main mechanism [33]. Wang *et al*. [34] found that a continuous infusion of high-dose (0.5 mg/kg) ketamine during surgery can significantly increase the levels of brain-derived neurotrophic factor and serotonin compared with low-dose (0.25 mg/kg) esketamine. A meta analysis showed that only high-dose esketamine (0.5 mg/kg) can improve PPD within 7 days and is more effective for PCIA than single use [35]. Yang *et al*. [36] found that the intravenous injection of esketamine (0.25 mg/kg) and its subsequent administration of 1 mg/kg or 2 mg/kg for PCIA can reduce PPD incidence at 7 and 42 days after surgery. Continuous intravenous infusion of 0.2 mg/kg esketamine can reduce PPD incidence at 4 [37], 7 and 42 days [38] after caesarean section. Therefore, we hypothesise that esketamine may have a dose-related antidepressant effect. Given that the side effects of esketamine are dose dependent, intravenous administration of single, large doses are unsafe and unethical for patients who underwent a caesarean section with effective spinal anaesthesia. Further research is needed to determine the optimal esketamine dose for improving PPD.

Subgroup analysis showed that esketamine might reduce PPD incidence in patients with an extroverted-stable personality, possibly because these patients experience less depression and anxiety and exhibit a stable and controlled emotional response. Therefore, an identical dose of esketamine reduced the PPD incidence in patients with this personality type. This finding is consistent with the study of Li *et al*. [39], who suggested that the effect of esketamine in improving PPD is related to prenatal characteristics and this drug is ineffective in women with pre-operative emotional instability and self-harm thoughts. However, no other relevant studies are available. Further research is needed to explore the specific mechanisms underlying these observations.

A statistical difference was observed among the personality types (introverted unstable > extroverted unstable > extroverted stable > introverted stable), and several potential mechanisms were associated with these differences. Firstly, significant differences in prenatal depression and pre-operative SAS and SDS scores were observed among individuals with different personality types. High scores were recorded for the introverted-unstable and extroverted-unstable personality types, and low scores were obtained for the extroverted-stable and introverted-stable personality types. Secondly, natural personality traits may contribute to this difference, with high introversion and neuroticism being strongly associated with PPD [40]. Generally, highly introverted individuals show a stable mood, dislike stimulation and prefer a stable and orderly lifestyle. Individuals with high neuroticism levels usually feel anxious, worried or depressed. Additionally, related studies revealed that neuroticism is associated with activity in the dorsomedial prefrontal lobe, whereas extroversion is associated with activity in the orbitofrontal lobe [41]. PPD is correlated with the activation of the amygdala, dorsomedial prefrontal cortex and orbitofrontal cortex [42, 43].

Regarding the safety of intravenous low-dose esketamine under spinal anaesthesia, we evaluated adverse effects, sedation scores, blood pressure and HR following administration. The esketamine-associated adverse reactions observed in this trial included dizziness, nausea, vomiting, hallucinations and nightmares. Blurred vision, diplopia and myalgia were not observed. No statistically significant differences in terms of intra- or post-operative adverse effects were found between the two groups. Although hallucinations and nightmares disappeared completely before the patients left the operating room, some of those in the esketamine group were able to clearly recall their content and perceived it as a worrying experience. These findings suggest that psychiatric symptoms associated with esketamine should be carefully considered prior to its clinical administration. Analysis of RSS >3 showed no statistical difference between the two groups 15 min after esketamine administration. Although some patients experienced drowsiness, all were awake when they left the operating room. Therefore, we did not assess post-operative sedation scores. MAP and HR were significantly higher in the esketamine group than in the control group, but both were within the normal range. On the basis of this assessment, a single intravenous injection of esketamine (0.25 mg/kg) during a caesarean section under spinal anaesthesia is safe and feasible but should be closely monitored to ensure the timely management of related complications.

## Limitations

This study had several limitations. Firstly, PPD was assessed on post-operative day 3, and long-term follow-up was not performed. Secondly, the main outcome indicators of this trial were subjective, and no objective indicators were included, which could have partially affected the accuracy of the outcomes. Thirdly, although this study found that the effects of esketamine differed among personality types, the underlying mechanisms were not investigated. Fourthly, the situation of the patients with each personality trait was not considered, and the small sample size of the study may have caused bias in the results. Fifthly, confounding factors were not excluded from the subgroup analysis; therefore, the findings should be considered exploratory. These deficiencies must be addressed in future studies.

## Conclusions

Our results showed that a single intraoperative application of esketamine does not decrease PPD incidence after caesarean section under spinal anaesthesia. However, it may reduce PPD incidence in patients with an extroverted-stable personality.

## Availability of Data and Materials

Data to support the findings of this study are available on reasonable request from the corresponding author.

## Supplementary Material

Supplementary material associated with this article can be found, in the online version, at https://doi.org/10.62641/aep.v53i4.1965.
