# Supplementary material for: Effects of Esketamine on Post-Partum Depression in Patients With Different Personality Types Undergoing Caesarean Section: Randomised Controlled Trial
Source: Actas Esp Psiquiatr. 2025 Aug 5;53(4):766–77. doi: 10.62641/aep.v53i4.1965 (PMC12353235; doi:10.62641/aep.v53i4.1965)
Supplement: Supplementary file 1 [file ActEsp-53-4-766-777-s1.zip › Supplementary Fig. 1.docx]

**Supplementary Fig. 1. Numeric rating scale scores during different time interval after surgery in patients with different personalities.** Numeric rating scale scores at rest (a) and on movement (b) in patients with an introverted-stable personality. Numeric rating scale scores at rest (c) and on movement (d) in patients with an introverted-unstable personality. Numeric rating scale scores at rest (e) and on movement (f) in patients with an extroverted-stable personality. NRS, numeric rating scale.
