# Supplementary figures and images for: Effects of Esketamine on Post-Partum Depression in Patients With Different Personality Types Undergoing Caesarean Section: Randomised Controlled Trial
Source: Actas Esp Psiquiatr. 2025 Aug 5;53(4):766–77. doi: 10.62641/aep.v53i4.1965 (PMC12353235; doi:10.62641/aep.v53i4.1965)

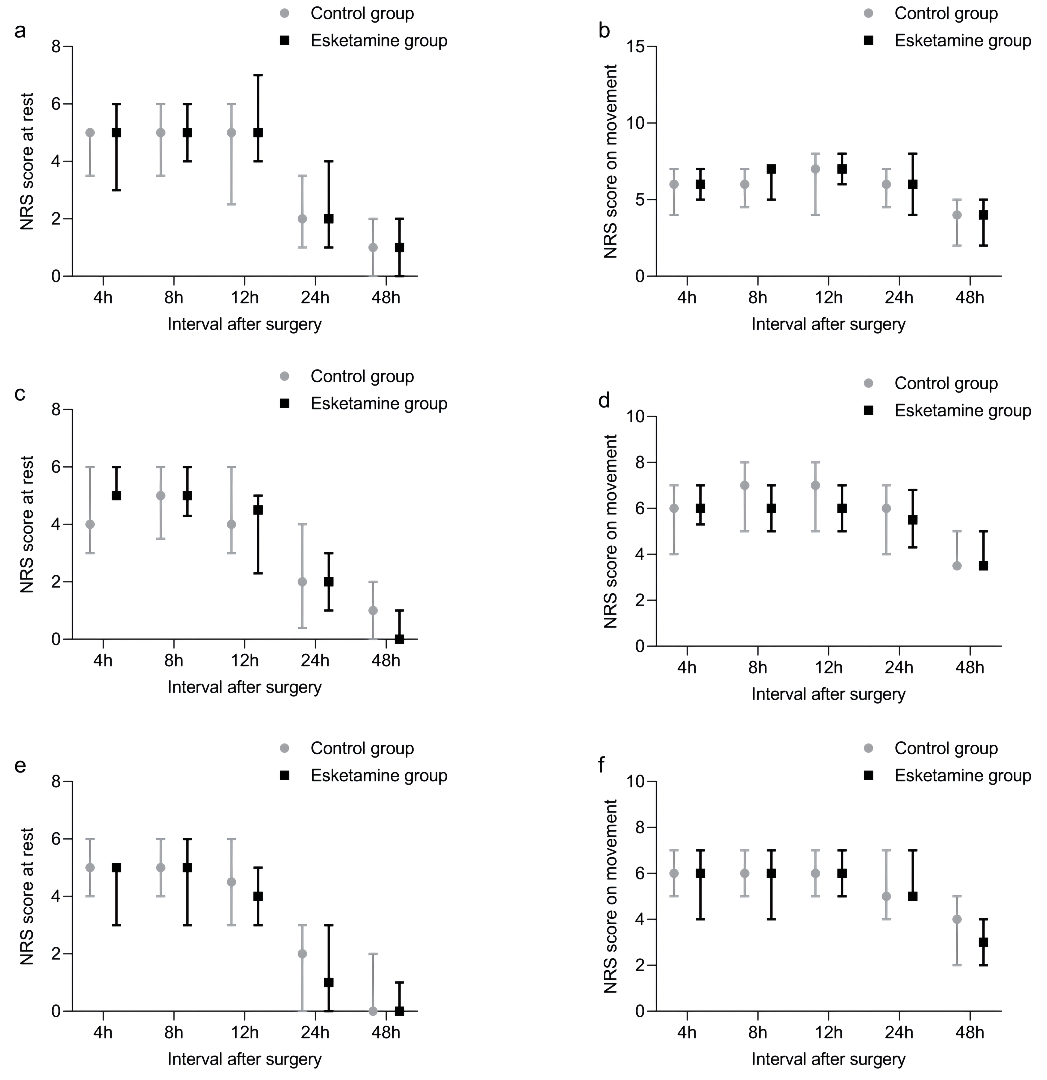

Supplement: Supplementary file 1 [file ActEsp-53-4-766-777-s1.zip › Supplemental material 1.png]
